# Experimental Study and ANN Dual-Time Scale Perturbation Model of Electrokinetic Properties of Microbiota

**DOI:** 10.3389/fmicb.2017.01216

**Published:** 2017-06-30

**Authors:** Yong Liu, Cristian R. Munteanu, Carlos Fernandez-Lozano, Alejandro Pazos, Tao Ran, Zhiliang Tan, Yizun Yu, Chuanshe Zhou, Shaoxun Tang, Humberto González-Díaz

**Affiliations:** ^1^Key Laboratory for Agro-Ecological Processes in Subtropical Region, Hunan Research Center of Livestock and Poultry Sciences, South-Central Experimental Station of Animal Nutrition and Feed Science in the Ministry of Agriculture, Institute of Subtropical Agriculture, Chinese Academy of SciencesChangsha, China; ^2^RNASA-IMEDIR, Computer Science Faculty, University of A CorunaA Coruña, Spain; ^3^Instituto de Investigación Biomédica de A Coruña, Complexo Hospitalario Universitario de A CoruñaA Coruña, Spain; ^4^Hunan Co-Innovation Center of Animal Production Safety, CICAPSChangsha, China; ^5^Institute of Biological Resources, Jiangxi Academy of SciencesJiangxi, China; ^6^Department of Organic Chemistry II, University of the Basque Country UPV/EHULeioa, Spain; ^7^IKERBASQUE, Basque Foundation for ScienceBilbao, Spain

**Keywords:** electrokinetic properties, zeta potential, artificial neural networks, perturbation theory, predictive model, ruminal microbiome

## Abstract

The electrokinetic properties of the rumen microbiota are involved in cell surface adhesion and microbial metabolism. An *in vitro* study was carried out in batch culture to determine the effects of three levels of special surface area (SSA) of biomaterials and four levels of surface tension (ST) of culture medium on electrokinetic properties (Zeta potential, ξ; electrokinetic mobility, μ_e_), fermentation parameters (volatile fatty acids, VFAs), and ST over fermentation processes (ST-a, γ). The obtained results were combined with previously published data (digestibility, D; pH; concentration of ammonia nitrogen, c(NH_3_-N)) to establish a predictive artificial neural network (ANN) model. Concepts of dual-time series analysis, perturbation theory (PT), and Box-Jenkins Operators were applied for the first time to develop an ANN model to predict the variations of the electrokinetic properties of microbiota. The best dual-time series Radial Basis Functions (RBR) model for ξ of rumen microbiota predicted ξ for >30,000 cases with a correlation coefficient >0.8. This model provided insight into the correlations between electrokinetic property (zeta potential) of rumen microbiota and the perturbations of physical factors (specific surface area and surface tension) of media, digestibility of substrate, and their metabolites (NH_3_-N, VFAs) in relation to environmental factors.

## Introduction

The rumen environment is characterized by a resident microbial population that rapidly colonizes and digests feed particles, thereby providing fermentation end products that are utilized by the host animal (Paracer and Ahmadjian, 2000). Enhancing feed digestibility by rumen microbiota is highly desirable for improving animal performance (Fox et al., 1995; Krause et al., 2003). Volatile fatty acids (VFAs) as a type of metabolites of feed fermentation in rumen providing metabolic energy for the microbial protein synthesis are directly absorbed by the host intestinal tract (Hungate, 1984). The physical-chemical properties of microbial cells and their environment play a vital role in microbial metabolic processes. Specifically, the specific surface area (SSA) of feed materials contributes to the biological processes of catalysis, adhesion, and digestion for microbes (Christensen et al., 1985; Shida et al., 2013; Yoda et al., 2014) as a vital factor of interface property (Garzón and Sánchez-Soto, 2015). Electrokinetic properties, such as zeta potential (ξ) and electrophoretic mobility (μ_e_), are highly relevant to this biological process (de Wouters et al., 2015). The ξ is quantified through measuring the μ_e_ of microbes under an electric field. The relationship between ξ and μ_e_ can be expressed as Henry equation (Hunter, 1981; Kaszuba et al., 2010) presented as Equation (1).

(1) $$\xi =\;\frac{3\mu_{\text{e}}\cdot \eta }{2\varepsilon_{\rho }\cdot \varepsilon_{\text{0}}}$$

Where ε_0_ represents the permittivity of free space, ε_ρ_ is the dielectric constant and η is the dynamic viscosity of dispersion medium, μ_e_ is the electrophoretic mobility and ξ is the zeta potential. The ξ represents the potential difference between the dispersion medium and stationary layer of fluid attached to the dispersed particle. It implies that ξ is a key indicator of the stability of colloidal dispersions. It has been proven that lower ξ absolute value means low viability in organism cells, which leads to the aggregation of cells in a dispersion medium (Kłodzińska et al., 2010).

In previous works, the experimental outcomes and predictive models have been reported based on perturbation values of cell physical properties (permeability and hydrophobicity) of *in vitro* rumen microbiota (Liu et al., 2016, 2017a). This study is focused on the perturbation effects on parameters such as ST, SSA, pH, c(NH_3_-N), VFAs and digestibility (D) of neutral detergent fiber (NDF) under the simulated rumen environment. However, no model has been reported yet to predict the ξ effect involved in time-dependent perturbations on these types of parameters during *in vitro* ruminal fermentation. One of the extreme difficulties, in this case, is that this is a very heterogeneous system with input variables that have perturbations over time measured on two different time scales.

In this line of thinking, ideas from the Perturbation Theory (PT) can be used to model the previously mentioned problem. The PT models are useful to find an exactly related and simpler solution to an existing sophisticated problem. The main feature of PT is to break the sophisticated issue into “perturbation” or “solvable” components. Our group has developed different PT models that start with a known solution to a problem and add corrections considering the variations of various experimental conditions (c_j_) (Gonzalez-Diaz et al., 2013). In the context of Cheminformatics, the effect of these deviations has been quantified using Moving Average (MA) operators. MA operators have been used to quantify perturbations in complex systems (Duardo-Sanchez et al., 2014), and nanoparticles (Luan et al., 2014; Messina et al., 2015).

This study is aimed at predicting ξ by fitting a linear model that uses the MA operators of all parameters along with different time scales as input values. In some cases, the linear model hypothesis may fail due to the high complexities of the natural system. In agreement with the previously mentioned concepts, some non-linear Machine Learning methods can be combined with time-series analysis methods, such as two of the most known examples of each area of Autoregressive Integrated Moving Average (ARIMA) and non-linear Artificial Neural Network (ANN). ARIMA and ANN algorithms have been compared to each other in time-series problems (Turias et al., 2008). However, the ARIMA and non-linear ANN can be further integrated to seek hybrid ARIMA–ANN models for different time-series issues (Babu and Reddy, 2014). In addition, the MA component of ARIMA models, developed by Box and Jenkins, also named the Box-Jenkins Operator, is basically the same operator that has been used to pre-process data previous to the ANN analysis (Box and Jenkins, 1968). In some previous works, a combination of ARIMA and ANN models was proposed in order to deal with data applying MA methods (Babu and Reddy, 2014), and to increase the prediction of time-series issues by using the smoothing strategies (Barba et al., 2014).

The present work focuses on the integration of ANN and ARIMA as models with PT operators to seek a new model able to predict the values of ξ (ζ*-potential*), given a set of perturbations on many input parameters measured on two different time scales. To this end, firstly new experimental measurements have been performed in terms of the perturbations on the values of ξ and μ_e_ of the rumen microbiota over time. Perturbations of different experimental physicochemical parameters such as SSA, ST, pH, D, c(NH_3_-N), VFA, and γ have also been measured over time. The present experimental data and the previous published data have been combined herein to develop a new predictive model over ξ in two-time scales (t_1_ and t_2_). ANN regression algorithms have been used with two types of variables as input variables. The first one was the expected measure (EM) of the ξ values for a given set of c_j_. The second type of variables was the MA operators of the experimental physicochemical parameters (for further details please see the following part). The best ANN time-series model accurately predicts the output ξ values for a set of 31,104 perturbations in a given set of c_j_ over a time span of 6–72 h.

## Materials and methods

### Experimental section

#### Methods and *in vitro* culture

The experiment was conducted to determine the effect of rumen culture medium at different ST levels and SSA of substrates on the electrokinetic properties of rumen microbes and fermented *in vitro* performances, the procedures of *in vitro* ruminal fermentation was described by our previous reference (Tang et al., 2008; Liu et al., 2016). In short, rumen fluid was equally obtained from 3 goats with rumen fistula before feeding, strained through 4 layers of cheesecloth, and mixed with an anaerobic fermented buffer in a ratio of 1:2 (v/v) under continuous flushing with CO_2_. The fermented buffer was prepared according to the description of Tang et al. (2006). About 500 ± 50 mg NDF extracted from rice straw was accurately weighed into a screw-cap serum bottle (145 mL) and full flushed with CO_2_. The fermented bottle, providing the inoculum of 50 mL mixed solution (39°C, pH = 6.9 ~ 7.0) of rumen fluid with buffer and 500 ± 50 mg DNF sealed with rubber stopper in full of CO_2_, was incubated and gently shaken in an incubator at the constant temperature of 39°C. As the limited of the experimental condition, we conducted the experimental in three times. For each time, we proceeded to all the treatments or combinational conditions (12 combinations = 4 ST × 3 SSA) at 6 or 4 fermented time-points (t_1_ = 6, 12, 24, 36, 48, and 72 h or t_2_ = 6, 12, 24, and 48 h after fermentation). The electrokinetic properties (μ_e_, ξ) of the ruminal microbiota and the conventional parameters were determined, along with the molar concentrations of VFAs and ST during fermentation (ST-a, or γ) in two different time series (t_1_ and t_2_). The parameters of μ_e_, ξ and γ were carried out on a time scale t_1_, while VFAs on a time scale t_2_. The experiment was conducted to measure these parameters under the conditions of 12 combinations with 4 ST (36, 43, 46, and 54 mN/m) × 3 SSA (3.27, 3.73, and 4.44 m^2^/g) with 3 replicates each. All experiments and animals used were provided by the Animal Care Committee, Institute of Subtropical Agriculture, the Chinese Academy of Sciences, China, with the document number (No. ISA-2012-018).

#### Construction of combinatorial conditions

In this study, an exogenous non-ionic surfactant, alkyl polyglucoside (APG) bought from Hunan Diyuan Co., Ltd., China, was provided to construct the different ST levels. The dosages of APG used in the present work were adjusted as described in our previous reports (Liu et al., 2013). The final ST levels were set to ST1, ST2, ST3, and ST4 with the values of 53.95, 46.09, 42.78, and 36.07 dynes/cm by supplying 0, 0.02, 0.05, and 0.12% (*v*/*v*) of APG, respectively. In addition, the percentages of APG used in the present study were similar to the ones from the current animal production (Yuan et al., 2010; Zeng et al., 2011). On the other hand, NDF extracted from rice straw was used as the substrate material for *in vitro* fermentation. According to rumen gastrointestinal digest particle distribution (Li and Jiang, 2001). NDF particles were ground into three different screen sizes with a grinder. The surface property of NDF particles expressed as specific surface areas (SSA1, SSA2, and SSA3) was determined by Surface Area Analyzer (Quadrasorb-SI, Quantachrome Inc. Florida, CA, USA), with the values of 3.37, 3.73, and 4.44 cm^2^/g, respectively.

#### Electrokinetic properties assay

In general, the fermentation microbial samples at each time-point were collected by centrifuging and washing with phosphate-buffered saline at least two times. The details for collecting microbial cells were described in our previous works (Liu et al., 2016). Briefly, fermented liquid (10 mL) from each bottle at the particular fermented time-point was centrifuged at 500 × rpm at 4°C for 10 min to remove the feed particles. The supernatant (2 mL) was then further centrifuged at 12,000 × rpm at 4°C for 10 min to collect the mixed microbial particles. The precipitate was full dissolved in 5 mL 4°C KNO_3_ with the concentration of 1 mM. The solution was centrifuged at 12,000 × rpm at 4°C for 10 min again, removed the supernatant, and then accurately diluted to 5 mL 4 C 1 mM KNO_3_ for 30 min. The solution (5 mL) was conducted to carry out the electrokinetic properties of RMMs assay. The electrophoretic mobility (μ_e_) of microbial cells was measured according to a phase amplitude light scattering (PALS) described by Hayashi et al. (Hayashi et al., 2003) and Eboigbodin et al. (de Kerchove and Elimelech, 2005), using the Zeta Potential Analyzer (NanoBrook ZetaPALS, Brookhaven Instruments Corp., Holtsville, NY, USA) (Instruments, 2012) equipped with a He-Ne laser (658.0 nm) as a source of light in a high precision system (30 cycles) at 39°C. The ξ was calculated according to equation (1) by the Zeta potential analyzer.

#### Surface tension during fermentation (γ)

The ST of inoculum in the fermentation process was determined on a time scale t_1_. The ST of the ruminal culture medium at each point time was measured immediately using the surface tension analyzer (model K100 Tensiometer, KRÜSS GmbH, Hamburg, Germany) (Blake et al., 1957). This ST was set in the actual fermentation processes as ST-a/γ.

#### Determination of volatile fatty acids

In current study, VFAs include acetic acid (C2:0), propionic acid (C3:0), butyric acid (C4:0), isobutyric acid (isoC4:0), valeric acid (C5:0), and isovaleric acid (isoC5:0). Every 2 mL of incubation fluid from each fermentation bottle on a time scale t_2_ were centrifuged at 10,000 × g and 4°C for 15 min. 1.5 mL of supernatant solution was collected, which was immediately mixed and homogenized with 0.15 mL 25% metaphosphoric acid. The mixture solution was centrifuged again at 10,000 × g at 4°C for 15 min, and the supernatant solution was collected to determine each VFA content by gas chromatography (HP5890, Agilent 5890; Agilent Technologies Co. Ltd, USA). The DB-FFAP column (Agilent, No.: 122-3232, 30 m in length with a 0.25 mm i.d. and 0.25 um thickness) was used. The parameters of this column were set as the attenuation in a nitrogen split ratio of 1:50, hydrogen flow 30 mL/min, airflow 365 mL/min, injector temperature 250°C, column temperature 150°C and detector temperature 220°C with N_2_ as carrier gas at a flow rate of 0.8 mL/min. The relative response factor, represented as the peak of each VFA, was calculated against a standard VFA mixture analyzed following every 10 measurements.

### Database construction and modeling

#### Original data resources

**Figure 1:**
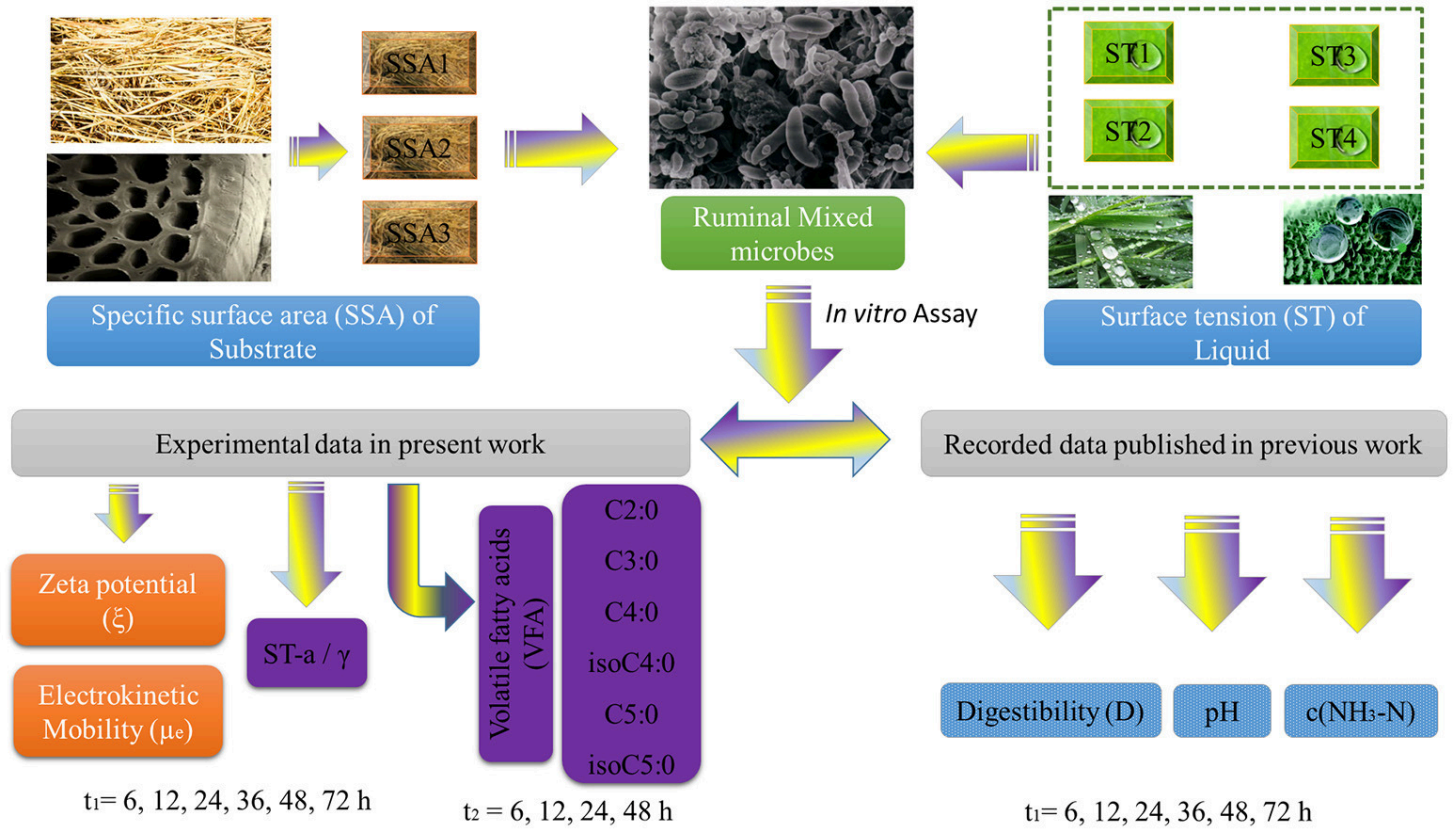
Flow chart of the experimental section used for the construction of the dataset.

In this work, the ξ and μ_e_ values of ruminal mixed microbes (RMMs), VFA and γ were obtained with the same initial combinatorial conditions of ST × SSA. The recorded data of pH, digestibility (D) of NDF, and c(NH_3_-N) were also collected from our previous works (Liu et al., 2016). All data came from two different time scale series (t_1_ and t_2_) to establish a predictive model. More specifically, for the variables of ξ, μ_e_, γ, D, pH, and c(NH_3_-N), the time series was t_1_. For the variables of VFAs (six individual VFA), the time series was t_2_. The details for all data resources used to develop the predictive ANN Time Series model are shown in Figure 1. The full dataset is provided online (Liu et al., 2017b) (SM01_Variables_t1.pdf, SM02_Variables_t2.pdf, and SM03_Zp_model_dataset.xlsx).

#### Details of dataset construction and modeling

The experiment considered the initial combinatorial conditions made up of 4 ST × 3 SSA = ST1-SSA1, ST1-SSA2 …ST4-SSA3 for all the variables, such as (a) experimental variables: ξ, μ_e_, γ, and VFA; (b) record data: pH, c(NH_3_-N), and D. However, the experimental variables were measured with a different time span of 6–72 h in t_1_ and 6–48 h in t_2_. More specifically, all variables combined with the time series can be expressed as ξ(t_1_), μ_e_(t_1_), ST-a(t_1_), pH(t_1_), c(NH_3_-N)(t_1_), and D(t_1_) in time series of t_1_; VFA(t_2_): C2:0(t_2_), C3:0(t_2_), C4:0(t_2_), isoC4:0(t_2_), C5:0(t_2_), and isoC5:0(t_2_) in time series of t_2_. Therefore, in order to study the effect of the perturbations of all variables over zeta potential (ξ) of RMMs, an integrated input dataset, made up of combinations of all the variables presented in these two different time scales (t_1_ and t_2_), needs to be assembled. There were two blocks of experimental data: (n_1_) No.(t_1_) = 12 initial c_j_ × 3 replicates × 6 point-of-time = 216 cases; (n_2_) No.(t_2_) = 12 initial c_j_ × 3 replicates × 4 point-of-time = 144 cases. In this work, an integrated dataset made up of No. = 31 104 (= 216 No.(t_1_) × 144 No.(t_2_)) cases was constructed, combining all variables in these two blocks of data on different time scales. The ξ value was standardized using the mean for all ξ. Next, the ξ-ANN models were developed to predict the numerical simulation ξ of rumen microbes over the environmental variables or factors in rumen fermentation ecosystem. For further details on n_1_ and n_2_ stocks experimental data (please see the online files SM01_Variables_t1.pdf, SM02_Variables_t2.pdf, SM03_Zp_model_dataset.xlsx, and SM04_Zp_dataset_std_values.sta) (Liu et al., 2017b).

#### ANN models

In this work, it was firstly assumed that the zeta potential of RMMs might associate with the environmental factors to some extent. Herein, it was assumed that a numerical model used to map the physicochemical properties of RMMs might be beneficial to reflect its mechanism or function. Therefore, the environmental factors such as pH, D, c(NH_3_-N), γ, and VFAs were taken into consideration. The variable symbols V_q_(t_1_) and V_f_(t_2_) were defined according to two different time scales, where the subscript “q” and “f” indicate the different input variables corresponding to the different time series, respectively. V_q_(t_1_) or V_f_(t_2_) indicates that the input variables change with the corresponding time scale. As the variability or the “*small*” change in different conditions c_j_, we introduced the deviations (perturbations) of each input variables based on the corresponding expected values (data dispersion). Therefore, the following types of terms were introduced. The first type is the variable < ξ>, which is the Expected Measurement (EM) component used to account for the expected value of the output property marked as ξ_(e)_, where the subscript “e” is the expected measurement. This theoretical notion has been widely used in our previous works (Ran et al., 2016). The other type refers to the Box-Jenkins Operators (or perturbation values) ΔV_ϕ_(t_k_), t_k_ representing the time scale t_1_ and t_2_, while V_ϕ_ is V_q_ or V_f_, respectively. In addition, the MA component was used to account for the dispersion of variables (Gonzalez-Diaz et al., 2013). In the present work, the terms < V_ϕ_(t_k_)> are the MA of the variable V_ϕ_(t_k_) in one of the original combinatorial experimental conditions with the format of 4 ST × 3 SSA.

(2) $$\Delta {\text{V}}_{\phi }\left({\text{t}}_{\text{k}}\right)=\;{\text{V}}_{\phi }\left({\text{t}}_{\text{k}}\right)-\langle {\text{V}}_{\phi }\left({\text{t}}_{\text{k}}\right)\rangle$$

In this sense, this is a complex theoretical model which combined the methods of the PT, MA and Time Series Analysis (Fisher, 1936). The general formula of the proposed model is shown as follows:

(3) $$\xi_{\text{pred}}=\;{\text{a}}_{0}+\xi_{\left(\text{e}\right)}+\sum_{\text{q }=\;1}^{\text{q }=\;{\text{q}}_{\text{max}}}{\text{b}}_{\text{q}}\cdot \Delta {\text{V}}_{\text{q}}\left({\text{t}}_{1}\right)+\sum_{\text{f }=\;1}^{\text{f }=\;{\text{f}}_{\text{max}}}{\text{c}}_{\text{f}}\cdot \Delta {\text{V}}_{\text{f}}\left({\text{t}}_{2}\right)$$

In this general formula, symbols such as a_0_, b_q_, and c_f_, are the intercept, or the coefficient of the corresponding variable. In this specific case, the ANN model is a linear additive function of all the terms mentioned above. The terms of ΔV_ϕ_(t_k_) were added as corrections to the EM of zeta potential ξ_(e)_ = a_1_. < ξ(t_k_)>, where a_1_ refers to the weighted value of < ξ(t_k_)>, and < ξ(t_k_)> refers to expected value of ξ calculated as the standardized value of ξ based on the mean value of each c_j_.

**Figure 2:**
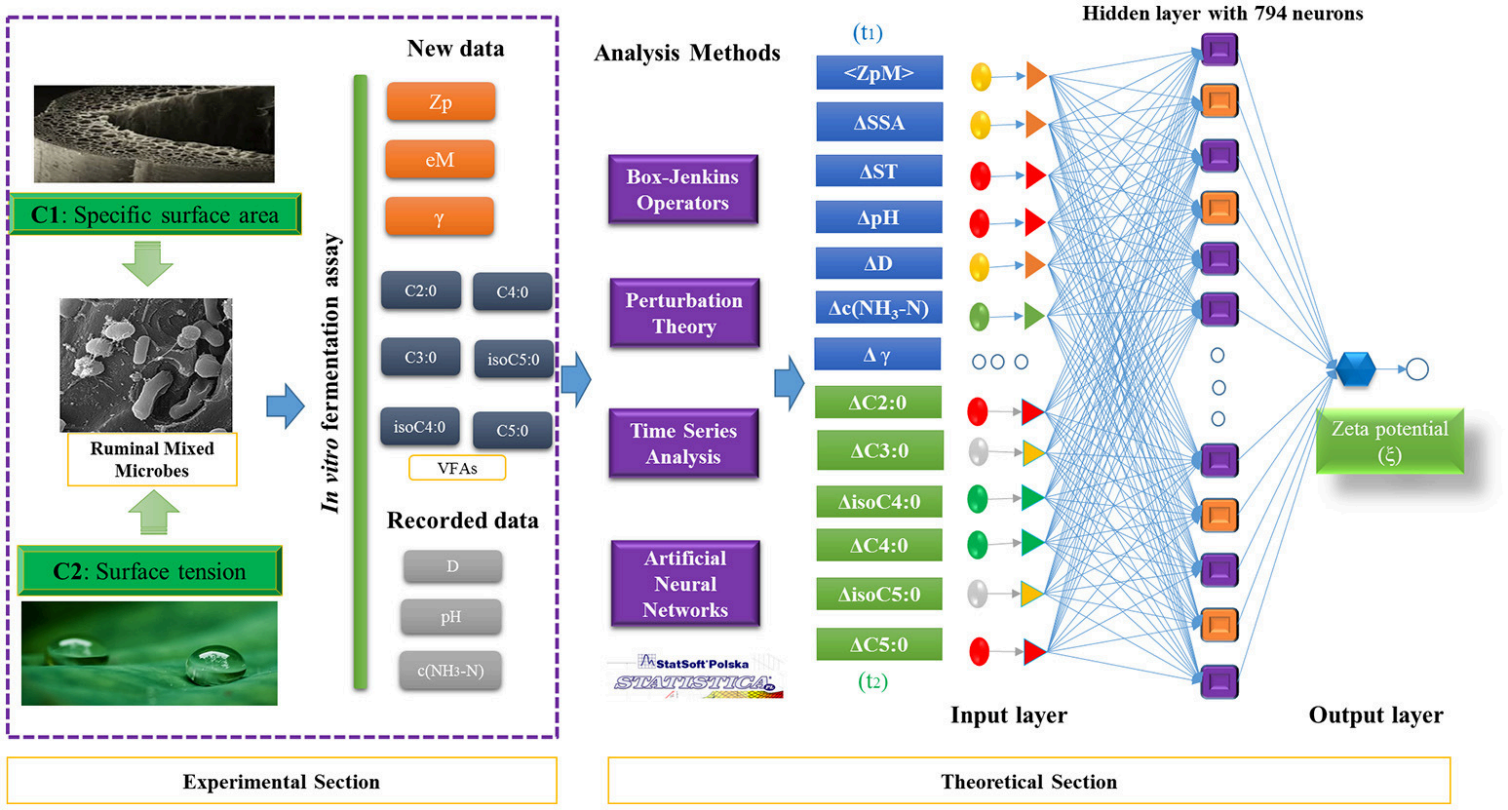
Flow chart of experimental and theoretical sections of this work.

Next, the expected measurements < ξ> and its corrections ΔV_ϕ_(t_k_) were obtained for the entire dataset. First the different ANN algorithms (Fisher, 1936) were trained/validated in a ratio of 3:1 (training/validation) to search for the best ξ-ANN model with STATISTICA software version 6.0 (STATISTICA, 2002). Three types of ANN algorithms were chosen to search for the best regression prediction model: Linear Neural Networks (LNN), Multilayer Perceptron (MLP), and Radial Basis Functions (RBF). In this sense, it was mandatory to use all factors, including the EM variable and all the corrections ΔV_ϕ_(t_k_), such as the input variables, in order to develop the predictive ANN regression models. In so doing, our group attempted to find the contribution weighted value of each variable in the zeta potential of RMMs. Thus, the back propagation with 100 epochs and Conjugate Gradient Descent with 500 epochs were used for retraining. The details of experimental and theoretical sections used to develop the predictive model for zeta potential were presented in Figure 2.

## Results

### Experimental study

The experimental results of ζ*-potential* (ξ), μe (μ_e_) and ST during fermentation (expressed as γ) in t_1_ time series are shown in Table 1. In order to understand the data errors in this table, we decided to include two different boxplots (for raw and standardized data) in the Figshare repository of this paper (Liu et al., 2017b). All the ζ*-potential* (ξ) values of microbes were ranged from −40 mV to −20 mV. In the present study, the ζ*-potential* (ξ) value was not affected by SSA, ST and their interaction (*P* > 0.05), while it changed along the incubation time (t) (*P* < 0.01) by the significance analysis. In addition, the effects of incubation time (t) on ζ*-potential* depended on its interaction with SSA of the substrate (SSA × time interaction, *P* < 0.05). For SSA at 3.37 m^2^/g, the increase of ξ from 12 to 24 h, and decrease from 24 to 48 h was greater than that of ζ*-potential* at 3.73 m^2^/g. The ST of rumen fluid could be decreased by adding a non-ionic surfactant, and a moderate decrease of ST could increase the permeability of the microbial membrane, which also was noted in the present study. The values in Table 1 are the mean of the three experiments.

**Table 1:** The experimental results of ξ and μ_e_ for ruminal microbiota cells in t_1_ (h) time series.

**SSA**	**ST**	**t_1_(h)**	**μ_e_**	**STD**	**ξ**	**STD**	**γ**	**t_1_(h)**	**μ_e_**	**STD^a^**	**ξ**	**STD**	**γ**
3.37	53.95	6	−3.12	−0.05	−31.92	−0.04	48.93	36	−2.38	1.79	−24.26	1.81	51.69
	46.09		−3.35	−0.67	−34.44	−0.66	49.41		−2.55	1.31	−26.23	1.32	50.39
	42.78		−3.44	−0.81	−35.29	−0.81	46.25		−2.72	0.98	−27.88	0.98	47.87
	36.07		−3.14	−0.39	−32.20	−0.38	39.87		−2.84	0.35	−29.13	0.36	45.02
3.73	53.95		−3.21	−0.61	−32.94	−0.60	47.53		−2.52	1.12	−25.86	1.11	50.20
	46.09		−3.06	−0.31	−31.41	−0.31	46.38		−2.70	0.59	−27.72	0.58	47.55
	42.78		−3.27	−0.51	−33.58	−0.51	46.58		−2.77	0.74	−28.42	0.74	47.85
	36.07		−3.44	−0.70	−35.27	−0.69	44.72		−2.94	0.53	−30.22	0.53	45.27
4.44	53.95		−3.22	−0.34	−33.02	−0.34	49.32		−2.72	0.89	−27.92	0.89	49.70
	46.09		−3.32	−1.01	−32.92	−0.76	48.91		−2.31	1.49	−23.76	1.45	48.48
	42.78		−3.48	−0.90	−35.71	−0.90	49.18		−2.54	1.44	−26.04	1.43	46.55
	36.07		−3.37	−0.62	−34.56	−0.62	44.43		−2.61	1.27	−26.82	1.25	44.71
3.37	53.95	12	−3.21	−0.29	−32.98	−0.30	52.01	48	−3.85	−1.88	−39.67	−1.91	47.25
	46.09		−3.25	−0.44	−33.40	−0.41	50.09		−3.81	−1.82	−38.90	−1.74	44.89
	42.78		−3.33	−0.54	−34.17	−0.54	47.59		−3.72	−1.50	−38.14	−1.50	44.06
	36.07		−3.22	−0.60	−33.12	−0.61	47.74		−3.67	−1.70	−37.61	−1.69	43.98
3.73	53.95		−2.94	0.06	−30.19	0.06	53.88		−3.23	−0.66	−33.19	−0.66	48.11
	46.09		−2.99	−0.13	−30.69	−0.13	49.52		−3.20	−0.66	−32.86	−0.66	46.40
	42.78		−3.20	−0.32	−32.80	−0.32	50.05		−3.32	−0.62	−34.09	−0.63	43.18
	36.07		−3.13	0.06	−32.18	0.06	45.05		−3.85	−1.72	−39.51	−1.71	41.96
4.44	53.95		−3.35	−0.66	−34.36	−0.66	51.21		−3.10	−0.05	−31.83	−0.05	46.33
	46.09		−3.09	−0.45	−31.77	−0.48	51.94		−2.92	−0.01	−29.94	−0.04	45.21
	42.78		−3.49	−0.93	−35.82	−0.93	49.04		−3.26	−0.37	−33.47	−0.36	43.56
	36.07		−3.13	−0.03	−32.10	−0.02	46.65		−3.37	−0.63	−34.62	−0.63	42.30
3.37	53.95	24	−2.70	0.98	−27.68	0.98	49.02	72	−3.31	−0.54	−34.01	−0.55	46.95
	46.09		−2.48	1.49	−25.47	1.50	46.44		−3.03	0.12	−31.71	0.00	45.34
	42.78		−2.41	1.74	−24.79	1.73	44.92		−3.06	0.14	−31.39	0.13	44.44
	36.07		−2.38	1.49	−24.48	1.48	44.03		−2.64	0.85	−27.11	0.85	44.18
3.73	53.95		−2.95	0.05	−30.26	0.05	50.16		−2.95	0.05	−30.25	0.05	45.17
	46.09		−2.67	0.66	−27.41	0.66	47.68		−2.99	−0.14	−30.71	−0.14	44.97
	42.78		−3.07	−0.01	−31.54	−0.01	46.01		−2.78	0.72	−28.47	0.73	44.21
	36.07		−3.06	0.24	−31.39	0.24	44.18		−2.52	1.58	−25.87	1.58	42.71
4.44	53.95		−2.79	0.73	−28.62	0.72	49.98		−3.31	−0.58	−33.99	−0.57	45.78
	46.09		−2.85	0.15	−29.61	0.04	47.20		−2.98	−0.16	−30.59	−0.20	45.65
	42.78		−2.97	0.36	−30.51	0.35	45.17		−2.96	0.40	−30.32	0.40	44.97
	36.07		−3.15	−0.08	−32.31	−0.07	45.21		−3.08	0.09	−31.64	0.09	44.60

a *STD represents the standardized values of ξ and μ_e_, respectively*.

The VFA values of the present study were shown in Table 2. In order to increase the interpretability of the information in this table, we decided to include two different boxplots (for raw and standardized data) in the Supplementary Materials of this paper (Figshare repository) (Liu et al., 2017b). The total VFA concentration and the molar percentages of most individual VFA were not affected by SSA, except for the molar percentage of propionate, which was increased (*P* < 0.05) with the increasing of SSA. By increasing the SSA of the substrate, the percent of propionate increased, while the ratio of acetate/propionate decreased. The ratio of acetate to propionate decreased (*P* < 0.05) with the increasing of SSA. Total VFA concentration and the molar percentage of isovalerate decreased (*P* < 0.01), but the molar percentage of propionate increased (*P* < 0.01) with increasing ST. By decreasing the ST of rumen inoculums, the percent of propionate decreased, but isovalerate increased, this phenomenon indicates that both of the SSA of the substrate and the ST of rumen fluid could change fermentation patterns of the fiber. The values in Table 2 are the mean of the three experiments. Thus, four plots (Raw_data_Errors_Table 1.png, Raw_data_Errors_Table 2.png, Standardized_data_Errors_Table 1.png, Standardized_data_Errors_Table 2.png) and an Excel file with the plot data (Data_errors.xlsx) were included in the Figshare repository (Liu et al., 2017b).

**Table 2:** The VFAs concentrations with the designated initial combinatorial conditions (3 SSA × 4 ST) in time series t_2_ (h).

**Initial conditions**	**Combinatorial**	**t_2_(h)**	**Concentrations (mM) of VFAs^a^**					
**SSA (m^2^/g)**	**ST (dynes/m)**		**C2:0**	**C3:0**	**C4:0**	**isoC4:0**	**C5:0**	**isoC5:0**
3.37	53.95	6	5.06	3.50	2.78	0.71	0.71	0.74
	46.09		6.94	3.90	3.37	0.59	0.59	0.93
	42.78		6.98	3.76	3.23	0.56	0.56	0.91
	36.07		6.29	4.10	3.58	0.62	0.62	1.05
3.73	53.95		4.25	3.45	2.79	0.58	0.58	0.81
	46.09		6.24	3.84	3.25	0.60	0.60	0.90
	42.78		4.96	3.50	3.30	0.59	0.59	0.96
	36.07		5.60	4.03	3.69	0.63	0.63	1.12
4.44	53.95		5.12	3.64	3.20	0.40	0.40	0.88
	46.09		5.94	4.00	3.63	0.57	0.57	0.99
	42.78		4.30	3.69	3.57	0.50	0.50	1.00
	36.07		5.86	4.16	3.85	0.59	0.59	1.11
3.37	53.95	12	6.57	4.56	3.58	0.41	0.41	0.96
	46.09		7.37	4.76	4.21	0.72	0.72	1.24
	42.78		8.80	5.06	4.43	0.70	0.70	1.27
	36.07		9.70	5.27	4.52	0.72	0.72	1.44
3.73	53.95		6.12	4.26	3.33	0.40	0.40	0.90
	46.09		5.49	4.30	3.70	0.64	0.64	1.10
	42.78		6.77	4.61	4.32	0.69	0.69	1.25
	36.07		9.49	5.30	4.60	0.72	0.72	1.40
4.44	53.95		5.07	3.91	3.02	0.51	0.51	0.83
	46.09		6.49	4.52	3.81	0.63	0.63	1.09
	42.78		7.85	4.71	4.02	0.65	0.65	1.14
	36.07		6.73	4.65	4.12	0.67	0.67	1.30
3.37	53.95	24	18.69	10.03	9.15	1.05	1.05	1.54
	46.09		17.97	9.68	9.68	1.20	1.20	1.73
	42.78		19.99	10.61	11.03	1.35	1.35	2.08
	36.07		17.38	8.68	9.30	1.25	1.25	2.24
3.73	53.95		17.74	9.93	8.92	1.20	1.20	1.55
	46.09		16.57	9.23	8.57	1.05	1.05	1.50
	42.78		22.18	12.13	12.07	1.47	1.47	2.22
	36.07		20.88	11.00	11.94	1.65	1.65	2.75
4.44	53.95		17.55	10.88	9.17	1.13	1.13	1.46
	46.09		20.38	11.18	9.33	1.18	1.18	1.57
	42.78		20.23	10.76	9.48	1.18	1.18	1.70
	36.07		18.43	9.14	8.54	1.21	1.21	1.77
3.37	53.95	48	13.18	8.10	6.48	1.05	1.05	1.69
	46.09		14.70	7.83	6.66	1.10	1.10	1.73
	42.78		14.91	8.76	7.80	1.25	1.25	2.05
	36.07		14.07	8.95	8.34	1.31	1.31	2.28
3.73	53.95		14.36	9.90	8.23	1.34	1.34	2.15
	46.09		11.74	8.57	7.29	1.15	1.15	1.86
	42.78		14.50	10.20	8.73	1.41	1.41	2.24
	36.07		12.70	8.71	6.97	1.10	1.10	1.77
4.44	53.95		14.24	9.96	7.27	1.11	1.11	1.77
	46.09		12.35	9.85	8.05	1.32	1.32	2.04
	42.78		17.22	11.78	10.04	1.59	1.59	2.52
	36.07		15.81	10.87	9.05	1.39	1.39	2.27

a *Where, C2:0 = acetic acid, C3:0 = propionic acid, C4:0 = butyric acid, isoC4:0 = isobutyric acid, C5:0 = valeric acid, and isoC5:0 = isovaleric acid*.

### ANN models

In the previous section, the output values of ξ (ζ*-potential*) for rumen microbiota were conducted to measure after the perturbations of the initial values of ST and SSA in this experiment. We separated all data into two sets of input variables, measured on two different time scales under the same conditions c_j_. In details, the physicochemical parameters *per se* and D were measured on the time scale t_1_ and the concentrations of VFAs on the time scale t_2_. Next, our group started to develop a general predictive model based on the data from the present work and from our previous studies (Liu et al., 2013). The ANN topologies tested included LNN, MLP, and RBF. The best model found was the RBF 14:14-794-1:1; which is able to predict the values of ξ for more than 30,000 cases with a correlation coefficient > 0.8, see details in Table 3. The models exported from STATISTICA can be downloaded as **SM05**_Zp.snn (Liu et al., 2017b).

**Table 3:** ANN Dual-time series models for ξ of rumen microbiota.

**ANN^a^**	**Dataset^b^**	**Correlation coefficient**	***SD***	**Error mean^c^**	**Error SD^c^**	**Training/Members^d^**
RBF 14:14-794-1:1	T	0.826	1.020	0.000	0.575	KM
	V	0.858	0.978	0.012	0.502	KN
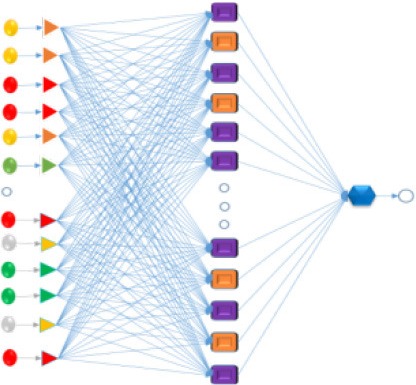						PI
MLP 1:1-5-1:1	T	0.273	1.020	−1.181	0.982	BP100
	V	0.000	0.978	−1.181	0.982	CG20
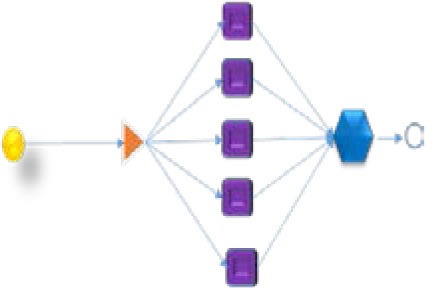						CG0b
LNN 15:15-1:1	T	0.349	1.020	0.000	0.956	PI
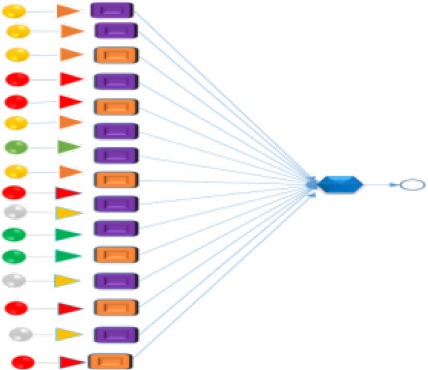	V	0.306	0.978	0.002	0.932	

a *LNN represents Linear neural networks, MLP is Multilayer perceptron, RBF refers to Radial basis functions*.

b *“t”: the training dataset; “v”: validation dataset, in a ratio of 3:1*.

c *Error mean = Errors of mean, and Error SD = standard deviations of errors*.

d *BP means Back Propagation, CG represents the Conjugated Gradient Descent, PI is Pseudo-Invert (Linear Least Squares Optimization), “b” refers to the best network, KM: K-means algorithm, and KN: K-nearest neighbor algorithm*.

Table 4 depicts the sensitivity ratio value (Var Ratio) for each input variable of the corresponding ANN model. Evidently, the value of the Var Ratio provides extremely important information for a predictive model; a greater value of Var Ratio provides a more important role in the predictive model. In addition, when the value is greater than 1, the corresponding variable plays an important role in the model, on the other hand, less than 1 indicates no or a little role for the model.

**Table 4:** RBF model sensitivity analysis compared to other models.

**ANN/Var Ratio**	**ξ_expt_**	**t_1_(h)**	**ΔSSA_1_**	**ΔST_1_**	**ΔD_1_**	**ΔC(NH_3_-N)_1_**	**ΔpH_1_**	**ΔST_1_-a**
RBF 14:14-794-1:1	1.00	2.03	1.54	1.27	1.90	1.15	1.00	1.03
MLP 1:1-5-1:1	1.02	—	—	—	—	—	—	—
LNN 15:15-1:1	1.00	1.08	1.00	1.00	1.07	1.02	1.13	1.04
ANN/Var Ratio	—	t_2_(h)	ΔC(Ace)_2_	ΔC(But)_2_	ΔC(Pro)_2_	ΔC(iBut)_2_	ΔC(iVal)_2_	ΔC(Val)_2_
RBF 14:14-794-1:1	—	1.20	1.19	1.19	1.28	1.12	1.15	1.12
MLP 1:1-5-1:1	—	—	—	—	—	—	—	—
LNN 15:15-1:1	—	1.00	1.00	1.00	1.00	1.7E+12	1.00	1.7E+12

Thus, for the case of the RBF model, all variables have sensitivity ratios >1. It shows that the perturbations on the variables measured on both time scales (t_1_ and t_2_) have a significant effect on the final value of ξ (ζ*-potential*) to some extent. In the context of physiological properties of RMMs, our best RBF model obtained further proof of the vital role of combinatorial conditions with the different levels of SSA and ST on the electrokinetic properties of RMMs. In addition, this new model showed that the fermentation time and digestibility play the most important role in zeta potential property of RMMs. However, the pH value and ST-a (γ) in the time series of t_1_ showed the lowest influence role in zeta potential of RMMs. The concentration of metabolites, such as ammonia nitrogen and VFAs, also plays an important role in this electrokinetic property for rumen microbiota. In particular, the role of the propionic acid is higher than that of other metabolites (NH_3_-N and other components of VFAs) for the ζ*-potential* of rumen microbiota in this case.

## Discussion

The cellulose degradation is highly correlated to the activity and quantity of fibrolytic enzyme. The previous work had proved that the inclusion of a non-ionic surfactant in the culture media resulted in a significant increase in the production of extracellular celluloses and β-xylosidase *in vitro* production (Long and Knapp, 1991). Despite the fact that rumen microorganisms can produce some bio-surfactants to decrease the surface tension of rumen liquor, but the decreased degree is limited (Liu et al., 2013). The results obtained from the *in vitro* study implied that the ST could be regulated to an optimum level for rumen microorganisms to grow better. On the other hand, the material surface roughness (in particular the specific surface area property), surface stiffness and softness are the other key factors influencing cell adhesion and behavior, such as the cellular morphology, proliferation, and phenotype expression (Engler et al., 2006; Chang and Wang, 2011).

The adhesion of ruminal microbiota to the substrate is the prerequisite for bacterial colonization and proliferation (McAllister et al., 1994). The electrokinetic properties (ζ*-potential* and μ_e_) are considered as the key factors in the adhesion of microorganisms on biomaterial surface (Ascencio et al., 1995). ζ*-potential* indicates the degree of repulsion between adjacent and similarly charged particles in the dispersion, and maintains the stability of the suspension or dispersion (Van der Biest and Vandeperre, 1999). The Zeta potential can be used as a parameter for characterizing the physicochemical properties of the bacterial cell envelope which are often used to characterize the adhesiveness of bacteria and biofilm formation (Cieśla et al., 2011). In the present study, it was found that rumen microbiota imparted negative ζ*-potential* values to keep the electrosteric stabilization from incipient instability (ζ*-potential* range from ±10 mV to ±30 mV) to moderate stability (±10 mV to ±30 mV) in suspensions at all SSA and ST levels (Hanaor et al., 2012). The cell surfaces of the rumen microorganism were negatively charged, which was consistent with the previous report (Delgado et al., 2005). It implies that the Gram-negative bacteria were the main microorganism in the medium (Zhang, 2008). In the present study, the ζ*-potential* of rumen microorganism unaffected by ST, which was inconsistent with the previous studies, while the ζ*-potential* changed with increasing bio-surfactant and non-ionic surfactant concentration for single cell or carbon black powers (Hua et al., 2003; Zhang et al., 2009; Cheng, 2014).

In the present study, the results also showed that the ζ*-potential* of rumen microorganisms increased with increasing fermentation time up to 24 h, and the increasing charge density on the microorganism surface decreased the resistance of microbe adhering to substrates (Li et al., 2014). The negative charge density of cell or bacteria were more adhesive to the positive-charged material (e.g., amine group-grafted polyethylene) compared to neutral- or negative-charged materials in the case of Chinese hamster ovary (CHO) cells, leukocyte adhesion, phagocyte migration, and osteoblast differentiation, etc. (Keselowsky et al., 2003; Chang and Wang, 2011). In addition, the incorporation of negative charges for cell/bacterial organisms may facilitate the adsorption of proteins which promote cell adhesion and responses (Thevenot et al., 2008) and promote alkaline phosphatase enzymatic activity (Keselowsky et al., 2005). In this study, the results may indicate that the main adhesion of microbiota to the substrates occurred within 24 h. In addition, the decreasing of the microbial ζ*-potential* after 24 h may be due to the decreasing of bacterial activity, or the agglomeration of bacteria as the population increasing. Soni et al. (2008) reported that the number and the shape of peaks obtained on electropherograms, which reflected the number of small aggregates originating from the individual or clustered bacterial cells, were negatively correlated with the zeta potential. Furthermore, in the late fermentation phase, dead bacterial cells may have also contributed to a lower zeta potential (Soni et al., 2008). In addition, the surface charge of bacteria was also significantly affected by the growth medium, bacterial age and bacterial surface structure (Delgado et al., 2005).

On the other hand, the VFAs are the secondary metabolites of rumen carbohydrate degradation. The iso-acids are required absolutely to stimulate the growth of several rumen bacterial species, particularly for the fibrolytic organisms (Allison, 1963; Bryant, 1973). Our results implied that the surface tension property of media and the SSA characteristic of the substrate could directly influence the composition and distribution of VFAs metabolites of rumen microbiota. This result is consistent with some other previous reports, such as the optimal surface tension value might increase the VFA production and microbial metabolic ability in the anaerobic fermentation of waste activated sludge by addition of bio-surfactants (surfactin, rhamnolipid and saponin) (Huang et al., 2015, 2016). In the fermentation of rumen microorganisms, the total VFA concentration was significantly higher with 2% Tween 80 than with no or 1% Tween 80 treatments (Kim et al., 2005). Our results showed that the molar percentage of propionate significantly increased with the ST of media, similar with the previous report by supplementation of Tween 80 (Kim et al., 2005).

In general, the PT method sets with an initial of a known exact solution for an issue or problem by adding the corresponding corrections due to the variations of different experimental conditions (Liu et al., 2015). This idea was widely used to solve many practical problems, such as the fatty acid distribution (Liu et al., 2015), goat growth yield via mRNA expression of ghrelin receptor and growth hormone receptor (Ran et al., 2016), carbon nanotubes (González-Durruthy et al., 2017), and drug-lymphocyte interactome networks (Tenorio-Borroto et al., 2016), etc.

In our previous studies, we reported that the ideas of PT combining with the ANN modeling were successfully used to predict the physic-chemical properties (permeability and hydrophobicity) of rumen microbes based on the combinatorial conditions of the ST and SSA in the processes of *in vitro* fermentation (Liu et al., 2016, 2017a). However, for another key factor of physic-chemical properties of rumen microbiota, the predictive model focusing on the electrokinetic property (ζ*-potential*, ξ) has not yet been reported. Thus, the present work focused on developing for the first time ANN models, which evaluate the variations of the ξ (ζ*-potential*) of the cells, present in the rumen microbiota due to perturbations of many different parameters on two different time scales. The best ANN model (RBF) for ζ*-potential* (ξ) of rumen microbiota was established with a correlation coefficient >0.8 for > 30,000 cases based on the input perturbations of the physicochemical parameters such as SSA, ST, pH value, digestibility of NDF, c(NH_3_-N) and VFAs for the dual-time scale. From a physiological view, the initial combinatorial conditions of the SSA of material and surface tension of media, as well as the fermentation time, digestibility of NDF and concentrations of metabolites (NH_3_-N, and VFAs) in fermentation process are highly related to the electrokinetic property (ζ*-potential*, ξ) of the rumen microbiota.

## Author contributions

YL, CZ, and ZT designed the research; YL, TR, YY, and ST conducted the research; YL, CM, CF, AP, and HG analyzed the experimental original data and constructed the predictive model; YL, TR, ST, CM, HG, and ZT wrote the full manuscript. All authors approved the final manuscript.

### Conflict of interest statement

The authors declare that the research was conducted in the absence of any commercial or financial relationships that could be construed as a potential conflict of interest.
